# Prof. Lefort on the Antiseptic Treatment of Wounds

**Published:** 1879-06

**Authors:** 


### Translations.

ARTICLE XII.

### PROFESSOR LEFORT ON THE ANTISEPTIC TREATMENT OF WOUNDS. Translated by Henry M. Lyman, M.D.

The following abstract of an address by the distinguished Professor of Operative Surgery in the Paris School of Medicine, delivered before the Surgical Society of Paris, is published in *Le Progrès Médical*, March 22, 1879. It will be read with interest by those who, during the past ten years, have followed the course of events in the domain of surgical practice without suffering the unquestionably brilliant results of the new methods to blind their eyes against the fallacy of certain theories which have been urged in explanation. The experiments of M. Pasteur, which are here noticed, are of a character to which allusion is hardly ever made by that greatly over-rated gentleman.

"Two theories are now before us. The first, which has been advanced by Mr. Lister, and which can be called the *theory of externality*, affirms that a wound becomes poisoned through contact with the air. It is therfore necessary that poisonous air should be excluded from the wound, and the mode of dressing devised for this purpose has been described as the antiseptic method. M. Lucas Championnière has gone further than Mr. Lister, and has spoken of *antiseptic surgery*.

"The second theory advances the hypothesis that wounds secrete a special virus, called sepsin by certain authors; that this virus may be absorbed, and may thus produce more or less serious accidents. I cannot wholly accept either of these theories; I am confident that in certain cases, under the influence of certain diathetic causes, constitutional conditions or bad hygiene, a wound may produce this poison, but that it will not occur in wounds treated under favorable circumstances. I also believe that in certain forms of so-called septicæmia — that indefinite general expression in which are merged so many differing conditions — contact with the air may be injurious. I think we may thus explain some of the phenomena of hectic fever. Clinically the fact seems to be demonstrated, and we know what precautions the surgeon adopts to prevent the entrance of air into a chronic cold abscess. But the general doctrine of M. Pasteur does not seem to me acceptable; it can be summarized in the following words: No germs, no suppuration. It is the germs, according to the theory, which produce the accidents; but, if so, since these germs are equally dispersed everywhere, why is the air of the hospital more noxious than that of the city? Why is the air of the city worse than that of the country? The wound is the same; if the air is the same, why should not accidents everywhere occur? I am very well aware that lately mention has been made of particular microbions. Davaine and Pasteur with remarkable adroitness and sagacity, have demonstrated that anthrax is due to a special proto-organism, and they have attempted to extend this theory to all infectious diseases. But the results have been negative. One of our professional masters has invited M. Pasteur into his hospital service, has placed under his charge two patients, of whom one was a victim of traumatic septicæmia, the other of purulent infection. M. Pasteur has made numerous investigations, but in neither case, either by direct examination or by cultivation, could he find bacteria.

"Besides, gentlemen, if we accept the theory of M. Pasteur, how shall we explain the success which attends the open method of of dressing? Roser, who succeeded Billroth at the Zurich hospital, has authorized the method of dressing without dressings; he places his stumps on cushions, without closing them, without protection of any sort, and his results have been better than those of Billroth, who practiced the method of close dressing. Wishing to have a clear idea regarding the dressing which the society of Moscow has recomended as the best, I employed it in a very serious case, a mechanic whose leg and thigh had been crushed by a locomotive. After making a double amputation, I employed the open dressing and achieved a very fortunate result. I do not believe in the germ theory.

"I know very well that a false theory may yet be associated with good practice, and I hasten to state that Lister's dressing is an excellent dressing, and that it has given me splendid results. I, however, do not believe that the success which we now enjoy can be wholly explained by the new method. We pay greater attention to hygiene than when, in 1855, I laid before the Surgical Society the first international statistics, and we were distressed to learn that the mortality in our hospitals was greater than the hospital mortality in London. We have remedied the difficulties to which I then called attention; our patients are better fed, and that simple fact has something to do with the improvement of our results. But people still die, and they will die in spite of Lister's own dressing. I see that his mortality in amputation of the thigh is 26 per cent. My own statistics are better than that, only 20 per cent. for the same operation, three deaths in fifteen amputations. And I could tell you that one of these patients died of exhaustion at the end of forty-six days; that a second, amputated by one of our internes, and cured, had a conical stump, for which I resected the end of the bone, and that for some unknown reason he died suddenly during the day of the operation; that the third patient died within an hour after the amputation, without rallying from the traumatic shock. I will not dwell upon these statistics, for you can find them fully related in the *Bulletin of the Academy of Medicine.*

"I have experimented with Lister's dressing, and have had magnificent success. In my opinion, it is the method which best and most quickly produces immediate union. I ascribe this fact to the causticity of carbolized solutions. For carbolic acid I have substituted alcohol and sulphate of zinc, and I have obtained exactly the same prompt recovery and immediate union as with Lister's dressing; the proof, therefore, seems clear to my mind. The action of these caustic substances, the immobility and compression of the stump, the deep union of the flaps, are the elements which in my opinion constitute a good surgical dressing. * * * I believe that a wound in good condition does not produce a septic matter which can poison the system; but I do believe that under the influence of certain conditions of defective alimentation, of constitutional diathesis, or of moral depression, a wound may produce such a poison, which may be transported by the air, and especially by the hands, by the dressing apparatus, the linen, the sponges, or the topical applications, and may thus infect other patients."

---

ALCOHOL AS AN ANTIDOTE FOR STRYCHNIA.—(M. Hameau, *Gaz. Méd. de Bordeaux and Journ. de Méd.*, Jan., 1879, p. 27.)

The author reports certain experiments undertaken to learn the action of subcutaneous injections of alcohol in case of strychnine poisoning. A rabbit poisoned in this manner, and in a state of apparent death for five minutes, was injected with a gram of alcohol of 90°; in less than three minutes the members were extended, and were no longer convulsed when irritated. In twenty-five minutes the animal was on its feet, had no convulsions spontaneous or provoked, and ate. The next day it was in perfect health. The same experiment renewed several times, gave the same result, while rabbits poisoned with strychnine and left to themselves, never recovered.

On the other hand, the same quantity of alcohol in a rabbit not poisoned, plunged the animal into a state of stupor, and caused death the next day.

The efficacy of alcohol injections in case of strychnine poisoning, would appear to be sufficiently demonstrated by these experiments. It remains to be shown, whether alcohol may be considered as an antidote to the poison itself, or as a powerful sedative of which the action upon the cerebro-spinal system is diametrically opposed to the action of the strychnine, and would be useful when there was a nervous erethism to combat, under whatever form it presented itself. This last opinion appears the most probable. The author thinks alcohol thus used may be of use in poisoning by strychnia; it might also in tetanus if one dared employ it in sufficient doses by the endermic method. However, in the only case in which the remedy was used, it failed, but the case was already *in extremis*.

